# Postural stabilization during bilateral and unilateral vibration of ankle muscles in the sagittal and frontal planes

**DOI:** 10.1186/1743-0003-11-130

**Published:** 2014-09-01

**Authors:** Noémie C Duclos, Luc Maynard, Joëlle Barthelemy, Serge Mesure

**Affiliations:** Aix-Marseille Université, CNRS, 7287, ISM UMR, 13288 Marseille France; CRF Valmante, UGECAM-PACA, 13275 Marseille France

**Keywords:** Proprioception, Plan-dependence, Postural control, Standing position, Unilateral vibration

## Abstract

**Background:**

The purpose was to investigate the postural consequences of proprioceptive perturbation of the Triceps Surae and Peroneus Longus muscles. These muscles are known to control posture respectively in the sagittal and frontal planes during standing.

**Methods:**

Standard parameters and the time course of center of pressure (CoP) displacements were recorded in 21 young adults, instructed to maintain their balance during tendon vibration. Following 4 s of baseline recording, three types of vibration (80 Hz) were applied for 20 s each on the Peroneus or Achilles tendons, either unilaterally or bilaterally (with eyes shut). The recording continued for a further 24 s after the end of the vibration during the re-stabilization phase. To evaluate the time course of the CoP displacement, each phase of the trial was divided into periods of 4 seconds. Differences between the type of tendon vibration, phases and periods were analyzed using ANOVA.

**Results:**

During all tendon vibrations, the speed of the CoP increased and a posterior displacement occurred. These changes were greater during Achilles than during Peroneus vibration for each type of vibration and also during bilateral compared with unilateral vibration. All maximal posterior positions occurred at a similar instant (between 12.7 and 14 s of vibration). Only unilateral Achilles vibration led to a significant medio-lateral displacement compared to the initial state.

**Conclusions:**

The effect of the proprioceptive perturbation seems to be influenced by the position of the vibrated muscle according to the planes of the musculoskeletal postural organization. The amplitude of the destabilization may be related to the importance of the muscle for postural control. The medial CoP displacement which occurred during unilateral Achilles vibration is not a general reaction to a single-limb perturbation. Proprioceptive input from the non-perturbed leg was not sufficient for the antero-posterior displacement to be avoided; however, it helped to gain stability over time. The non-perturbed limb clearly plays an important role in the restoration of the postural referential, both during and immediately following the end of the vibration. The results demonstrated that at least 16 s of vibration are necessary to induce most postural effects in young adults.

**Electronic supplementary material:**

The online version of this article (doi:10.1186/1743-0003-11-130) contains supplementary material, which is available to authorized users.

## Background

The role of the postural control system is to continuously manage the body’s state of equilibrium in order to avoid falls. Balance is constantly perturbed by body and limb motion and the postural control system must select and integrate relevant sensory information in order to maintain postural control[1]. Upright postural behavior is comparable to an inverted pendulum[2]. The perturbation of one source of sensory information will result in different consequences depending on the pertinence of that information for the postural requirements at that instant[3]. Postural control in the sagittal plane predominantly occurs at the ankle[4], with the Soleus muscle playing a major role in keeping the body upright and controlling antero-posterior oscillations[5]. In contrast, in the frontal plane, postural control mainly occurs at the hip level[6]. However, it has been shown that control also occurs at the ankle, with the Peroneus Longus providing lateral stability[7]. During normal upright standing, proprioceptive information from the legs provides the most pertinent source of afferent information for the control of postural sway[8]. Most studies of proprioception around the ankle joint have focused on the sagittal plane (for example[9, 10]) and the frontal plane has been little explored. This is probably because of the mechanical stability naturally provided by the distance between the two feet in normal standing[11]. Nevertheless, a deficit of proprioceptive capacity could explain the medio-lateral instability[12] typically found in older adults, which is considered to be the best predictor of the risk of falls[13].

Many studies have used sudden movements of the supporting surface to explore the role of the ankle in postural reactions in both planes of motion[14]. There are two problems with the use of this paradigm: (i) the amplitude of destabilization often generates hip strategies rather than ankle strategies and (ii) the perturbation is applied bilaterally whilst the postural challenges which occur during daily activities are often unilateral. Thus, in order to better understand the role of the ankle in postural stability, it is necessary to explore the role of proprioception (i) during both unilateral and bilateral perturbations (ii) from ankle muscles which act predominantly in the sagittal and frontal planes. Tendon vibration is a useful tool for this purpose because it can be used to perturb proprioception in specific muscles[15]. The vibration principally activates primary (Ia) afferents in the muscle spindles[16], mimicking muscle stretch[15]. When the vibration is applied at 80 Hz[17] to a subject in quiet standing, the vibration generates an afferent signal[18] and a postural reaction occurs to ‘restore’ muscle length and avoid the illusory fall. The amplitude of the postural reaction reflects the integration of this signal in the postural scheme.

Most studies have evaluated the effect of bilateral Achilles vibration (for example[19, 20]), showing that it results in a backward shift of the CoP. The few studies which have applied Achilles vibration to a single limb[21, 22] have shown that a medial displacement of the CoP also occurs. However, the underlying mechanisms have not been explored. The medial displacement could be a general response to the perturbation of a single limb, irrespective of the tendon targeted (Achilles or Peroneus longus). Moreover, during bilateral vibration, the CoP shift gradually reduced as the vibration continued[20]. Questions however remain regarding the time course of the CoP displacement as well as regarding the specific role of the non-vibrated limb. It remains to be determined whether its role is to counteract the effect of the vibration or to regulate the postural reaction.

The aims of this study were threefold. The first aim was to determine if the postural reaction depends on the plane of motion controlled by the perturbed muscle. The second aim was to determine the role of the non-perturbed limb as a function of the direction of the induced reaction. The third aim was to investigate the time course of the CoP displacement during and following the perturbation in the different vibration conditions.

## Methods

### Participants

21 young-adults (11 women/10 men; 24.0 *(4.83)* [mean *(SD)*] years old, 69.3 *(10.8)* kg and 1.75 *(0.08)* m; dominant hand: 17 right/4 left; dominant eye: 14 right/7 left; dominant foot: 8 right/13 left) were included. All participants signed an informed consent form prior to the study. The study was conducted in accordance with the Declaration of Helsinki and approved by the Local Ethics Committee *Sud-Méditerranée II* (n°ID-RCB 2012-AOO518-35).

### Apparatus

**Figure 1:**
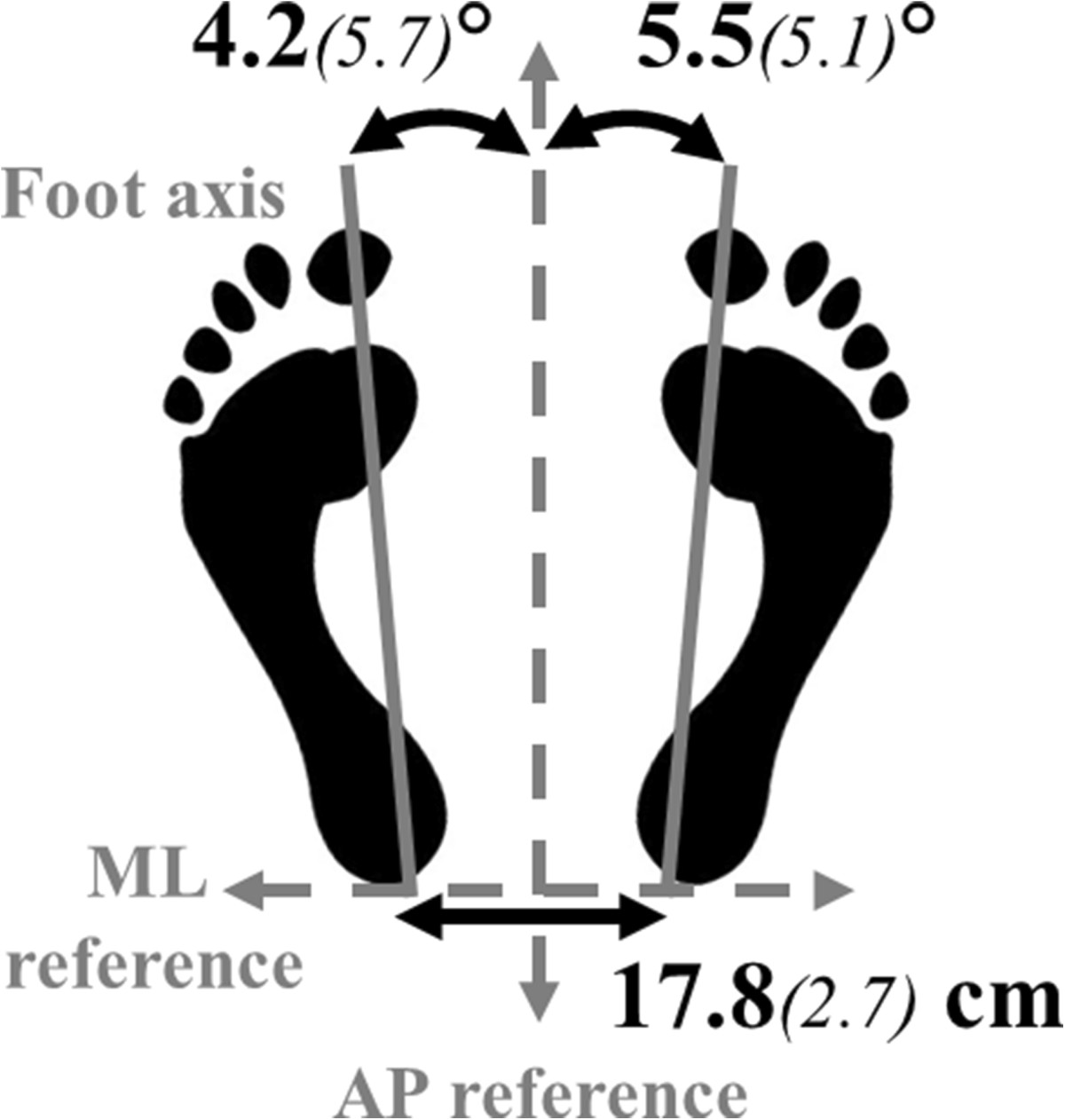
**Mean group parameters of the foot position naturally adopted along the ML and AP axes.** Feet were oriented along the AP reference of the platform with a mean angle which was not significantly different between feet. Heels were spontaneously aligned along the ML reference (0.0*(1.1)* mm), and were separated by the distance classically reported in the literature for preferred foot position[23].

Each subject was asked to stand quietly barefoot on the force platform, with their feet in a natural position (Figure 1;[23]) and one foot on each side of the AP axis reference mark on the force plate. Vibrations were carried out on the tendons of two muscles: peroneus longus (just above the lateral malleolus, with additional manual palpation;[24]); and gastrocnemius-soleus (at the level of the lateral malleolus). Subjects were instructed to maintain their balance at all times.

### Experimental protocol

Foot positions were marked on the force plates in order to ensure constant placement throughout the experiment. Three types of vibration (each repeated twice) were carried out: left unilateral, right unilateral and bilateral. Site order and trial sequences were randomized. Subjects wore opaque glasses to block vision for all trials. They were asked to keep their gaze in a straight-ahead direction. Unless they needed to rest, the subjects did not remove the opaque glasses for the duration of the experiment.

No vibration was applied for the first 4 seconds of each trial (P1 phase); vibration was then applied for 20 seconds (P2 phase;[25]) and the recording was continued for a further 24 seconds after the end of the vibration during re-stabilization (P3 phase). A DC motor with an eccentric load on the shaft, embedded in a plastic tube which was 7 cm long and 2,5 cm wide (VB 115, TechnoConcept, Mane, France), was used. Vibration frequency was set at 80 Hz with an amplitude of 0.2-0.5 mm[17]. These parameters are considered to be the most pertinent for the generation of illusory movement according to Roll et al.[15–17].

### Data acquisition

Ground reaction forces were calculated from 8 vertical mono-axial dynamometric load sensor cells; at a frequency of 40 Hz. The acquisition of the ground reaction forces was synchronized with the stimulus. Force data were then processed using Matworks’s MATLAB v.6 software to calculate postural parameters.

### Postural parameters

Antero-posterior (AP) and medio-lateral (ML) displacement of the CoP were recorded. The following parameters were then calculated for each phase of each trial:

Speed of the CoP displacement, calculated as total excursion of the COP divided by the duration, in each phase.

Mean anterio-posterior and medio-lateral CoP positions (mm); Negative values represent a posterior or left position relative to the center of the platform.

Maximum and minimum positions (mm) along the antero-posterior axis (AP-max and AP-min) and their time of occurrence (s).

To compare positions across trials, the mean position of the CoP at P1 was centered on zero for both axes by subtracting its coordinates from all the data samples recorded throughout the trial.

### Time course of the CoP

To evaluate the time course of the CoP displacement, each phase of the trial was divided into periods of 4 seconds. The vibration phase, P2 (20 s) was divided into 5 periods (P2_1_ through P2_5_). The last phase, P3 (re-stabilisation, 24 s) was divided into 6 periods (P3_1_ through P3_6_).

### Statistical analysis

After verifying the normality of the data, all parameters were analyzed using a general linear model repeated measures of variance analysis (ANOVA). Differences between the Achilles and Peroneus tendon vibration were analyzed using ANOVA with three within-subject factors: “Tendon” (Achilles, Peroneus), “Type” (bilateral, right unilateral, left unilateral) and “Phase” (P1, P2, P3). Postural adaptations relative to the initial state and over time were analyzed for each tendon using ANOVA with two within-subject factors: “Type” and “Period” (P1/P2_1_-P2_5_, then P1/P3_1_-P3_6_). A parametric Newman-Keuls post hoc test was used to determine the locus of differences. An α level of 0.05 was used for all tests.

## Results

All subjects accomplished the task without falling. There were no significant differences between conditions for the parameters analyzed during the P1 phase. In the text, “unilateral vibration” refers to both right and left unilateral vibrations, unless otherwise specified.

### Effect of the tendon vibrated

There was a *Tendon × Type × Phase* interaction for all parameters (Table 1). CoP displacements were perturbed during the vibration and also when it stopped, depending on the tendon and the type of vibration.

**Table 1:** **ANOVA results for both Achilles and Peroneus tendon vibrations**

Parameters	Tendon effect	Type effect	Phase effect	Tendon × Type × Phase interaction
Speed	**F(1,20) = 54.2**	**F(2,40) = 104.0**	**F(2,40) = 2.8**	**F(4,80) = 7.2**
	***S***	***S***	***S***	***S***
AP position	**F(1,20) = 10.3**	**F(2,40) = 8.0**	**F(2,40) = 80.5**	**F(4,80) = 18.2**
	***S***	***S***	***S***	***S***
ML position	F(1,20) = 0.9	**F(2,40) = 16.0**	F(2,40) = 0.7	**F(4,80) = 17.0**
	p = 0.35	***S***	p = 0.5	***S***

ANOVA results showing the effect of ‘Tendon’ (Achilles/Peroneus Longus), ‘Type’ (bilateral/left unilateral/right unilateral), ‘Phase’ (P1: stable/P2: vibration/P3: restabilisation) and their interactions. Significant results are written in bold text. *S* = statistically significant (p < 0.05).

### Vibration phase (P2)

**Figure 2:**
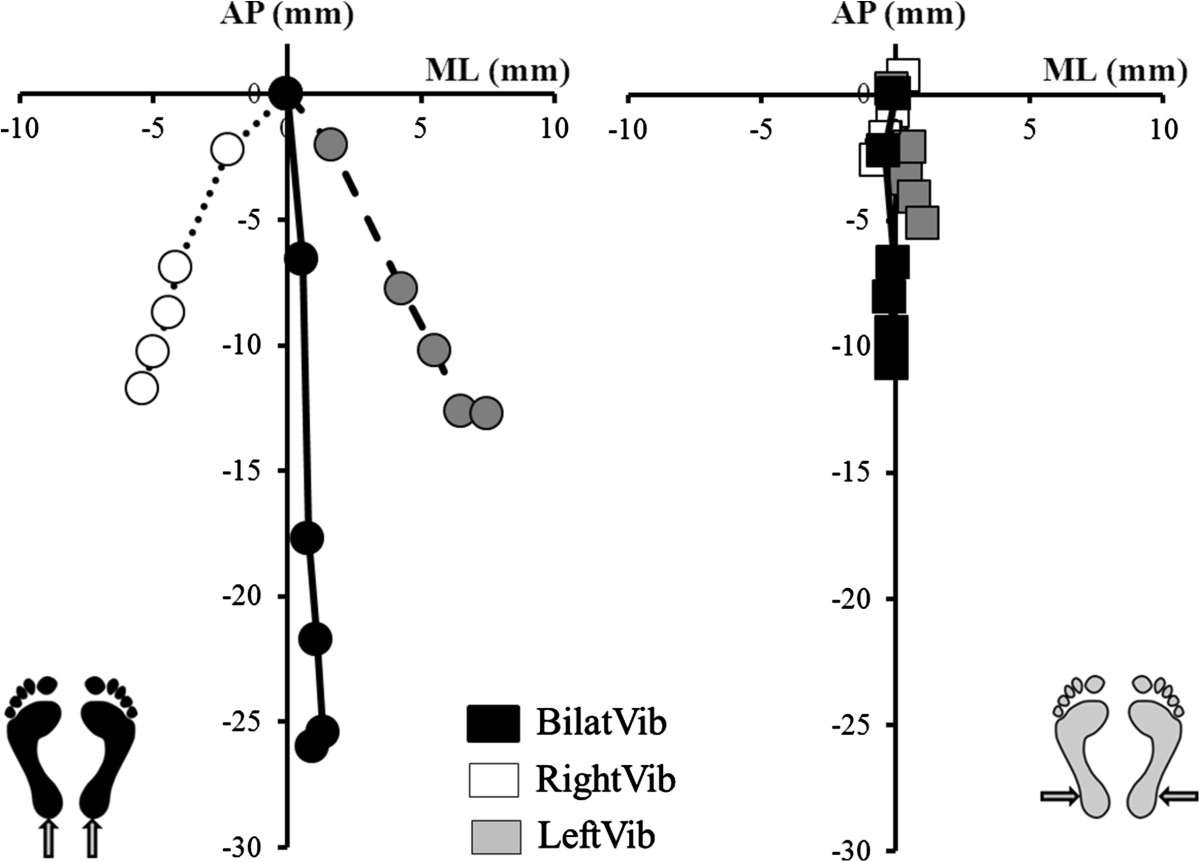
**Antero-posterior and medio-lateral positions during Achilles and Peroneus vibration.** Representation of AP (antero-posterior) and ML (medio-lateral) coordinates of the mean positions for the 5 periods of the vibration-phase (P2), during [left] Achilles (circles) and [right] Peroneus (squares) vibration, bilateral (black squares); right unilateral (white squares) and left unilateral type (grey squares). ML displacement was significant for unilateral Achilles vibrations only.

All tendon vibrations led to an increase in the speed and a posterior displacement of the CoP. This effect was significantly greater during Achilles than Peroneus vibration (p < 0.01) for each type. For both tendons, the speed and the posterior displacement were larger during bilateral than unilateral vibration (p < 0.01; Figure 2). Only unilateral Achilles vibration led to a significant ML displacement compared to the initial state (Figure 2).

**Figure 3:**
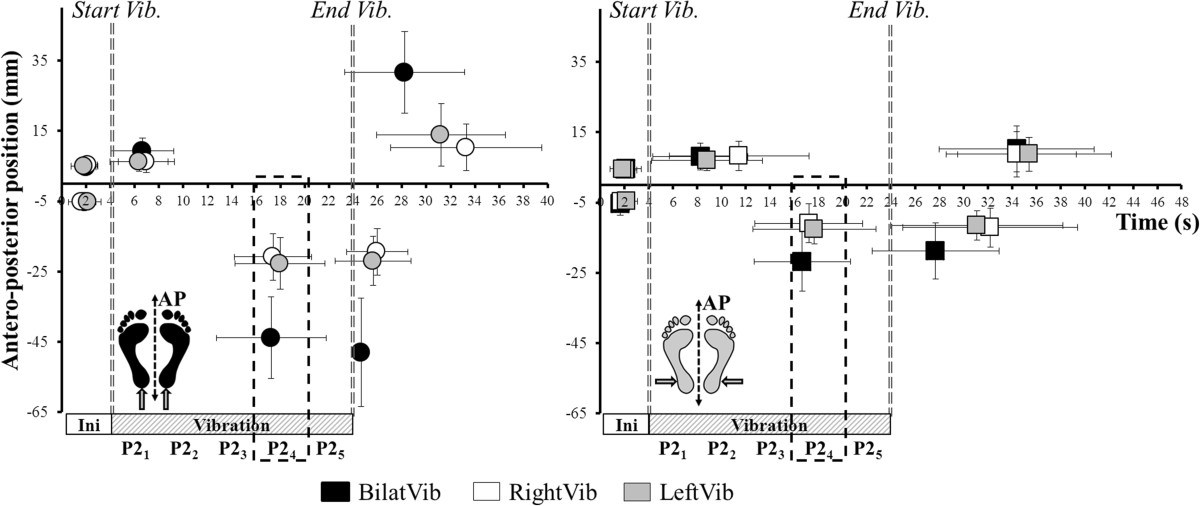
**Extreme maximal and minimal antero-posterior positions during Achilles and Peroneus vibration.** Representation of extreme (min/max) antero-posterior positions during [left] Achilles (circles) and [right] Peroneus (squares) vibration, during bilateral (black); right unilateral (white) and left unilateral (grey) type of vibration. The delimited zone represents 12–16 s of vibration. All the Y-min positions occurred during this period.

The AP-max position (anterior to zero) was similar for all conditions of vibration. The AP-min position was significantly more posterior for Achilles than Peroneus vibration (p < 0.01 for each condition) and it was also more posterior for bilateral than unilateral vibration (p < 0.01; Figure 3). The AP-min position during right and left unilateral Achilles vibration was respectively 49.42% *(18.22)* and 54.38% *(22.64)* of the AP-min during bilateral vibration. The AP-min position during right and left unilateral Peroneus vibration was respectively 51.76% *(20.55)* and 63.74% *(23.87)* of the AP-min during bilateral vibration.

### Time course of the CoP during vibration (P1/P2_1_ – P2_5_)

There was a main effect of *Type* and *Period* on mean CoP position along the AP axis during both Achilles and Peroneus vibrations and also a *Type × Period interaction* (Table 2).

**Table 2:** **ANOVA results for Achilles and Peroneus tendon vibrations separately**

Parameters	Type effect (P1/P2_1_– P2_5_)	Period effect (P1/P2_1_– P2_5_)	Type × Period (P1/P2_1_– P2_5_) interaction	Type effect (P1/P3_1_– P3_6_)	Period effect (P1/P3_1_– P3_6_)	Type × Period (P1/P3_1_– P3_6_) interaction
**Achilles vibration**						
Speed	**F(2,40) = 54.9**	**F(5,100) = 36.3**	**F(10,200) = 10.3**	**F(2,40) = 80.6**	**F(6,120) = 114.1**	**F(12,240) = 38.9**
	***S***	***S***	***S***	***S***	***S***	***S***
AP position	**F(2,40) = 62.4**	**F(5,100) = 114.5**	**F(10,200) = 26.9**	**F(2,40) = 9.6**	**F(6,120) = 6.8**	**F(12,240) = 4.6**
	***S***	***S***	***S***	***S***	***S***	***S***
ML position	**F(2,40) = 45.3**	**F(5,100) = 3.0**	**F(10,200) = 35.7**	**F(2,40) = 9.60**	F(6,120) = 0.8	**F(12,240) = 7.6**
	***S***	***S***	***S***	***S***	p = 0.5	***S***
**Peroneus vibration**						
Speed	**F(2,40) = 35.9**	**F(5,100) = 13.8**	**F(10,200) = 6.1**	**F(2,40) = 12.9**	**F(6,120) = 24.8**	**F(12,240) = 7.6**
	***S***	***S***	***S***	***S***	***S***	***S***
AP position	**F(2,40) = 23.8**	**F(5,100) = 23.8**	**F(10,250) = 10.7**	F(2,40) = 0.7	**F(6,120) = 2.6**	F(12,240) = 1.5
	***S***	***S***	***S***	p = 0.5	***S***	p = 0.1
ML position	F(2,40) = 0.7	F(5,100) = 0.1	F(10,200) = 1.6	F(2,40) = 0.2	F(6,120) = 0.3	F(12,240) = 0.6
	p = 0.5	p = 1	p = 0.1	p = 0.8	p = 0.9	p = 0.9

ANOVA results carried out for each ‘Tendon’ (Achilles/Peroneus Longus) showing the effect of ‘Type’ and ‘Period’ for phases P2 and P3 of the trial and their interactions. Significant results are written in bold text. *S* = statistically significant (p < 0.05).

**Figure 4:**
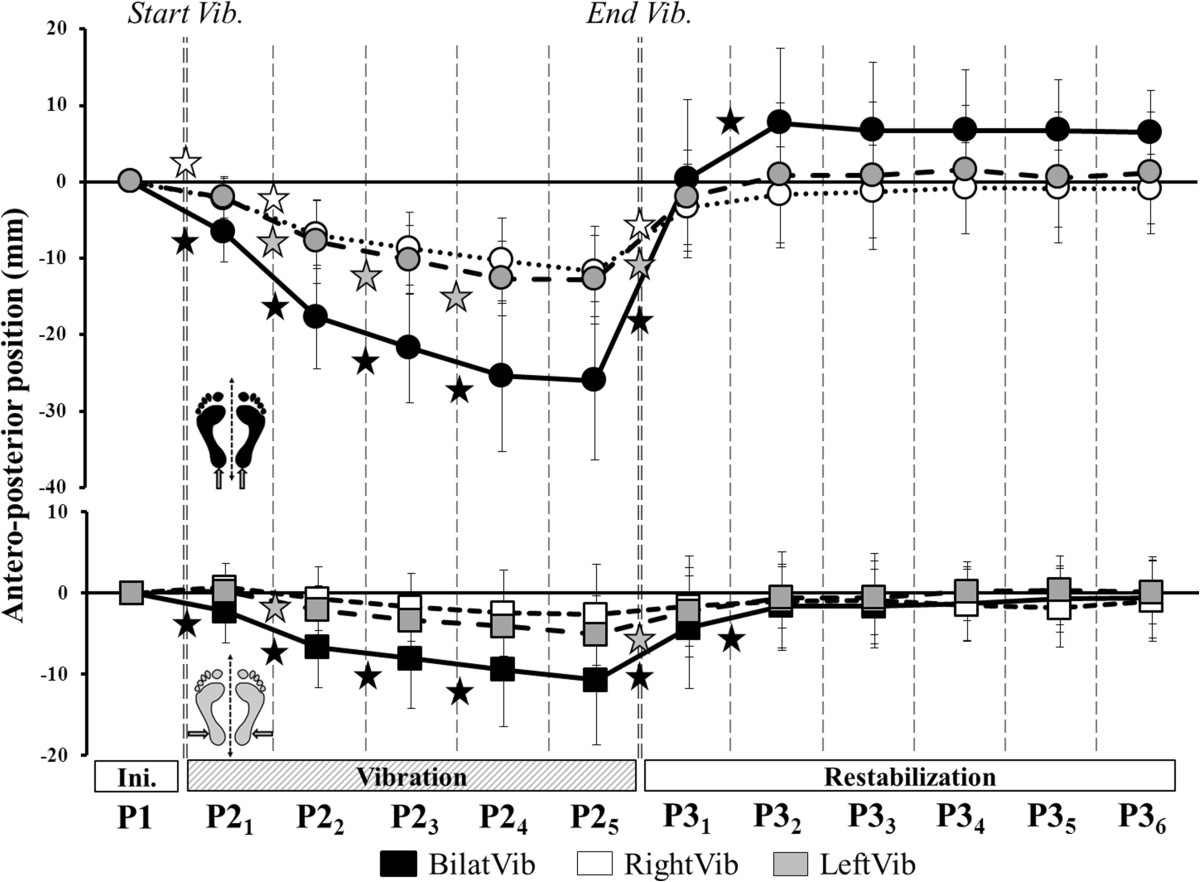
**Antero-posterior displacements during Achilles and Peroneus vibration.** Time course of the AP-position across all periods of the trial during Achilles (top, circles) and Peroneus (bottom, squares) vibration. Stars denote a significant change in position between successive periods of the same phase.

Mean CoP position shifted significantly posteriorly from P2_1_ through P2_4_ for bilateral and unilateral left Achilles vibration (Figure 4)_,_ then it stabilized. For unilateral right Achilles vibration, the mean position only shifted significantly posteriorly from P2_1_ to P2_2_.

Mean CoP position also shifted significantly posteriorly from P2_1_ through P2_4_ for bilateral Peroneus vibration. During unilateral left Peroneus vibration, the mean CoP position only shifted posteriorly from P2_1_ to P2_2_ and during right Peroneus vibration there was no change from the mean P2_1_ position.

**Figure 5:**
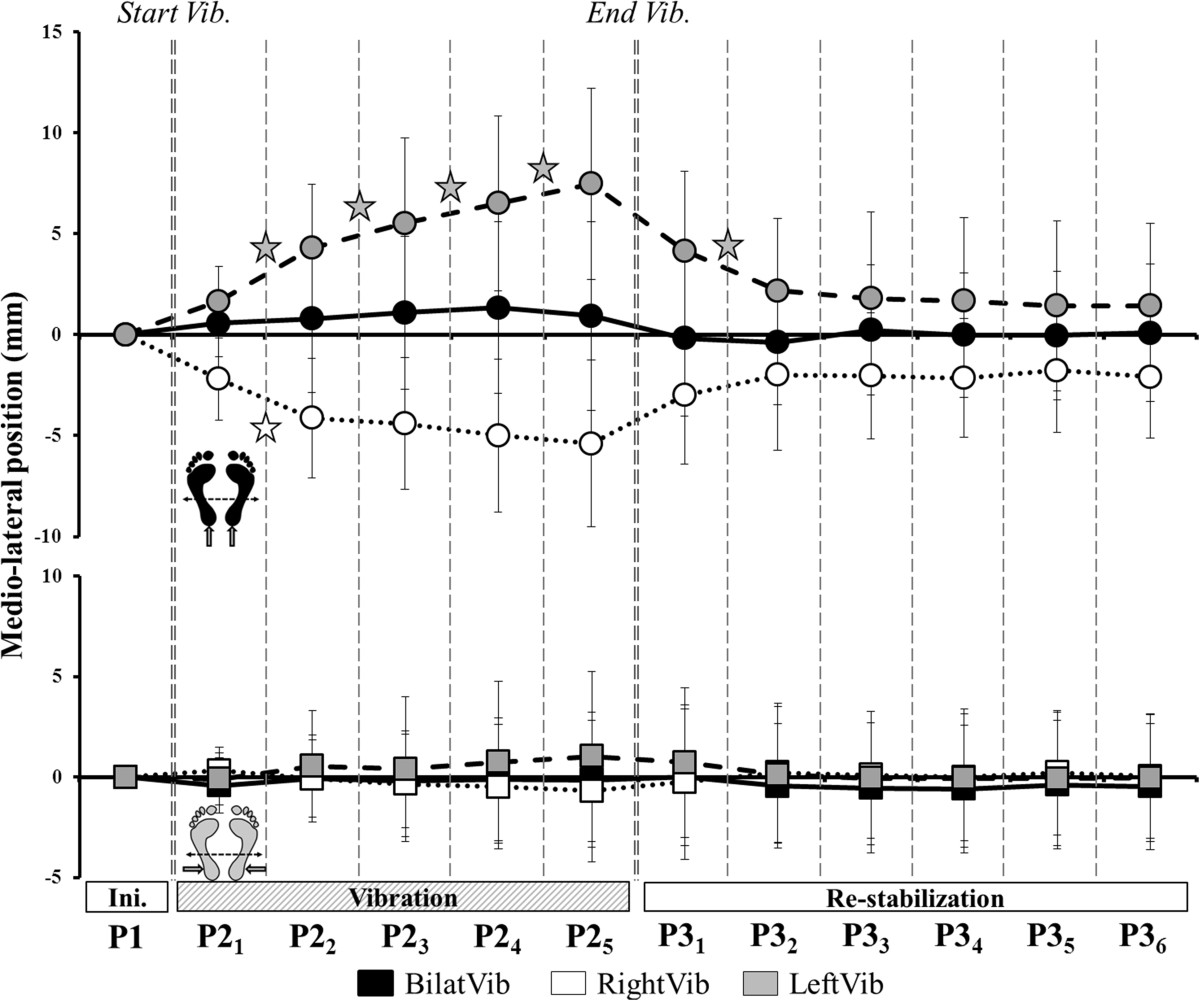
**Medio-lateral displacements during Achilles and Peroneus vibration.** Time course of the ML-position across all periods of the trial during Achilles (top, circles) and Peroneus (bottom, squares) vibration. Stars denote a significant change in position between successive periods of the same phase.

There was a main effect of *Type* and *Period¸* and a *Type × Period interaction* for the mean medio-lateral CoP position during Achilles vibrations only (Table 2). The position shifted significantly medially across all the periods of P2 during left unilateral vibration whereas it stabilized from P2_2_ in the right unilateral vibration (Figure 5).

**Figure 6:**
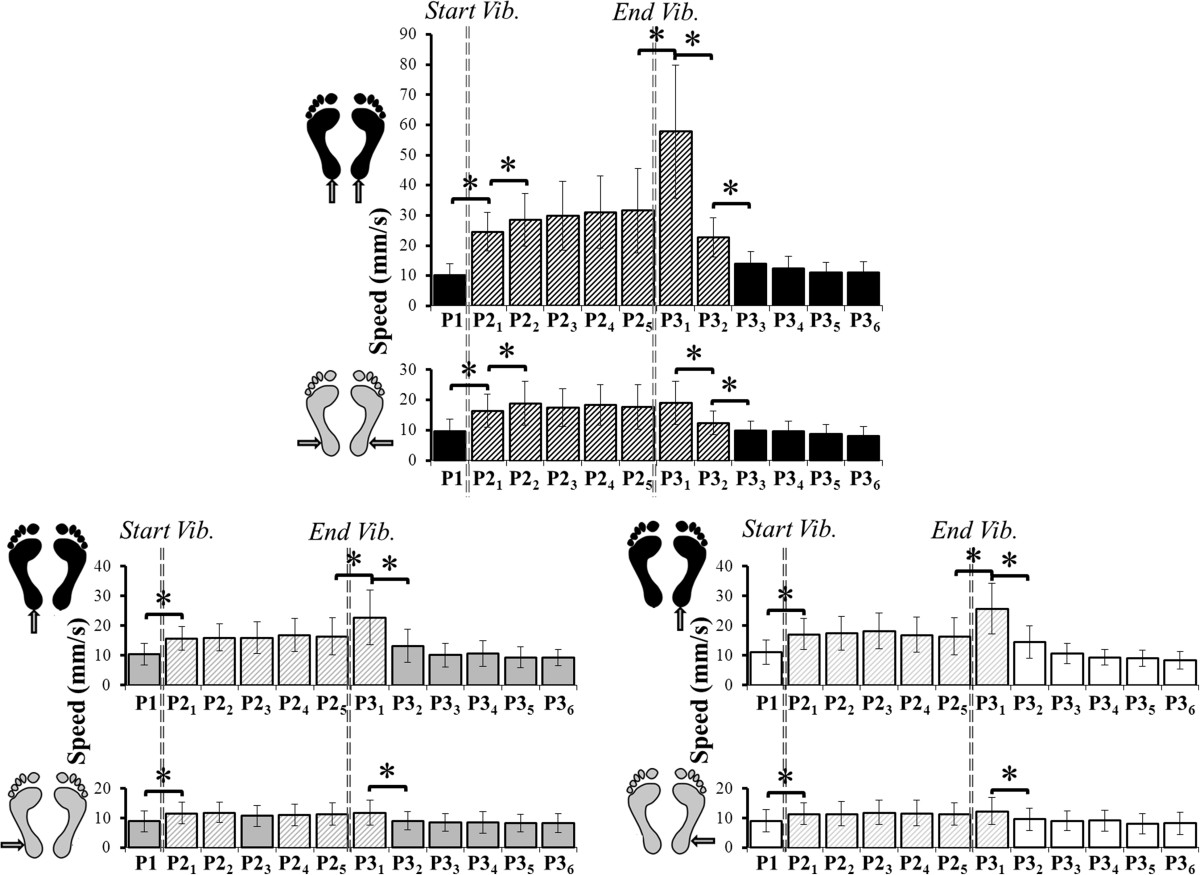
**Speed of the CoP displacement during Achilles and Peroneus vibration.** Change in the speed of the CoP during all periods for Achilles and Peroneus vibration. Top: bilateral vibration (black). Bottom left: left unilateral vibration (grey). Bottom right: right unilateral vibration (white). Stars denote a significant difference in mean speed between periods. Hatched bars are significantly different to the initial state (P1).

There was a main effect of *Type* but no effect of *Period* (Table 2) on CoP speed during both Achilles and Peroneus vibration. For bilateral Achilles and Peroneus vibrations, the speed of the CoP increased significantly from P1 to P2_1_ and from P2_1_ to P2_2_ (Figure 6). For all unilateral Achilles and Peroneus vibrations, the speed increased from P1 to P2_1_ only, then stabilized until the end of vibration.

The AP-max occurred at a similar instant (between 2.35 s and 4.8 s of vibration) for all conditions of vibration (except for right unilateral Peroneus). All AP-min positions occurred at a similar instant (between 12.7 and 14 s of vibration, ie. in the P2_4_ period) regardless of the tendon or type of vibration (Figure 3).

### Post-effect of vibration (P1/P3_1_ – P3_6_)

P3 postural parameters were not averaged because they were affected by the irremediable return to the initial state. The parameters were thus analyzed by periods (P3_1_ through P3_6_). There was a main effect of *Period* (Table 2) for all parameters except the ML-position of the CoP. After bilateral Achilles vibration, the mean AP-position remained anterior to zero until the end of the trial. In contrast, after cessation of unilateral Achilles vibration, the CoP returned to its initial position on the AP-axis almost immediately. Mean ML-position, however, differed from zero until P3_3_ after left Achilles vibration and until the end of the trial after right vibration.

After all Peroneus vibrations, mean AP-position returned close to zero at P3_2_ and mean ML-position did not differ from zero, as was the case during the vibration phase.

Regardless of the tendon vibrated, the speed of the CoP returned close to the initial state at P3_3_ after bilateral vibration and at P3_2_ after unilateral vibration (Figure 6).

Extreme positions occurred very soon after the end of the vibrations (Figure 3). Cessation of vibration led to a maximal posterior position (AP-min), which was more posterior and occurred earlier after Achilles than Peroneus vibration. A maximal AP-position then occurred which was more anterior and earlier after Achilles than Peroneus vibration.

## Discussion

### Does the postural reaction depend on the plane controlled by the vibrated muscle?

The bilateral proprioceptive perturbation of both the Achilles and Peroneus tendons induced postural reactions in the sagittal plane but not in the frontal plane. All unilateral perturbations also induced reactions in the sagittal plane. Postural reactions occurred in the frontal plane only during unilateral Achilles vibration. The systematic antero-posterior reaction is in accordance with the functional roles of the muscles stimulated. The Triceps Surae muscle is massively implicated in the control of movement in the sagittal plane[5]. The role of Peroneus Longus in this plane has been little described but was expected since an illusory movement with a sagittal component has previously been demonstrated following vibration with the foot free[16]. These results show that, in order to maintain the system’s requirements for balance and orientation in the sagittal plane, postural adjustments occurred immediately following the onset of the proprioceptive perturbation[26]. In this plane, the human musculoskeletal system has multiple degrees of freedom which have large ranges of motion[26]. It is very difficult for the postural system to counteract the effect of a proprioceptive perturbation without the use of visual input which usually stabilizes antero-posterior oscillations[27]. The amplitude of the destabilization may be related to the functional importance of the muscle in postural control. In the frontal plane, joint mobility is much more restricted[28] and the lower limbs form a closed-chain mechanical system[29] creating large biomechanical constraints. This could explain the lack of a significant medio-lateral displacement during the bilateral stimulation of both tendons. Some studies which used a constrained (such as toe-to-heel[30] which is highly sensitive to ML instability) or standardized foot position found a medio-lateral displacement in response to unilateral vibration on the lateral face of the ankle[3]. In the present study, the choice of a natural foot position could have created some mechanical medio-lateral stability[11]. In the normal standing position evaluated, the proprioceptive perturbation of the Peroneus was not sufficient to induce a medio-lateral displacement, despite the fact that it is directly implicated in the control of frontal plane motion. In contrast, the results showed that unilateral proprioceptive vibration of the Triceps Surae muscle always induced a medio-lateral displacement, despite the natural (stable) position of the feet. Thus, the medial displacement which occurred during unilateral Achilles vibration was not a general reaction to a single limb perturbation. The effect of the proprioceptive perturbation therefore seems to be influenced by the position of the vibrated muscle according to the planes of the musculoskeletal organization for normal standing control.

### Is the role of the non-perturbed leg influenced by the direction of the induced movement?

The non-perturbed leg was not always sufficiently effective to ensure stability in the frontal plane. The postural system may adopt a voluntary or possibly reactive strategy, involving the transfer of body weight to the non-vibrated limb in order to avoid the perturbing effect of the vibrations. Nevertheless, the results of this study showed that proprioceptive input from the non-perturbed leg did not completely prevent the antero-posterior displacement induced by vibration. However, analysis of the time course of the CoP displacement showed that its position was quickly stabilized. The halting of the CoP displacement may reflect a central phenomenon of sensory reweighting based on input from the non-vibrated limb. This process appeared to be effective for antero-posterior postural control but it failed to stabilize the medio-lateral axis. This highlights a paradox in the control of the different planes of body motion. Proprioceptive input from the non-perturbed leg did not prevent antero-posterior displacement but helped to gain stability over time; however, it did prevent medio-lateral displacement. Nevertheless, if medio-lateral displacement began to occur, the postural system could not stop it. Multi-directional postural control is particularly complex in bipeds[31] and the postural system appears to be ineffective beyond a certain level of difficulty. The results of this study suggest that the level of difficulty is determined by the plane of the perturbation.

During tendon vibration, the CNS constructs a new sensory reference frame in order to maintain balance in spite of the illusion of falling forwards and the misperception of verticality[32]. When the proprioception of both Achilles tendons is simultaneously perturbed, the only available source of information regarding body position is vestibular. However, vestibular information is insufficient to restore the gravitational vertical without visual input if proprioceptive input is non-congruent. When the vibration stops, the initial postural referential must be restored. The initial antero-posterior position of the CoP was immediately restored after unilateral vibration of both muscles but not after bilateral Achilles vibration. The duration of the post-effect after bilateral vibration is, however, subject to discussion in the literature and appears to be highly subject-dependent[19, 33]. In the present study, a post-effect was still present 25 s after the end of the 20 s of bilateral vibration. Thompson et al.[19] did not find any post-effect 25 s after the end of 30 s of bilateral vibration whereas Wierzbicka et al.[33] found some post-effects of bilateral vibration which persisted for up to 1 h in some subjects. However, a new finding of the present study was there was no significant post-effect after unilateral vibration. Thus, the non-perturbed limb clearly played an important role in the restoration of the postural referential, and this occurred as soon as the vibration ended. The results again showed the influence of the plane of motion controlled by the perturbed muscle. The position of the CoP remained shifted towards the contralateral limb following unilateral Achilles vibration. It could be hypothesized that (i) postural control in the frontal plane is not sufficiently accurate to detect this weight-bearing asymmetry; (ii) the postural abilities of young adults permit them to deal with this asymmetry without a risk of falling; (iii) the preceding perturbation may enhance a natural tendency for limb load asymmetry, suggested to be a functional adaptation for example to facilitate a step[32].

### Does the proprioceptive perturbation continue to disrupt the system in spite of postural reorganization?

Schmid et al.[34] describe the process of adaptation to a perturbation of balance as the achievement of a steady-state, despite an ongoing perturbation. Antero-posterior displacement of the CoP during bilateral Achilles vibration is the main indicator of postural adaptation. Postural reactions which occur during vibration are not just the sum of local reflex adjustments[25] but are also the result of central changes. The position of the CoP was stable during P2_4_-P2_5_ (12-16 s and 16-20 s of vibration). Its position during these periods was similar to the position recorded by Thompson et al. between 27.5 and 30 s[19]. This adaptive postural response may be an attempt to control the loss of stability caused by the multi-segmental displacement in response to the illusory movement. However, the antero-posterior stabilization also occurred following Peroneus vibration, despite the fact that the antero-posterior displacement of the CoP was smaller (less than that which occurred after 4-8 s of bilateral Achilles vibration). The assumption of mechanical stability does not explain this 2nd observation. The maximal posterior positions of the CoP occurred at similar times for all the vibration conditions (around 12-16 s of vibration), irrespective of the degree of disequilibrium. This is in accordance with the results of Schmid et al.[34] which showed that the adaptation process does not depend on the type of perturbation but rather on the time elapsed from the beginning of the perturbation. McKay et al.[22] found a similar phenomenon in children: the level of sensory-motor maturation affected the amplitude of the displacement induced by vibration, but not the time of occurrence of the maximal displacement. To our knowledge, studies which have analyzed the time course of postural effects only considered one type of vibration (either unilateral or bilateral). Capicíková et al.[20] showed that the mean posterior displacement of the CoP during bilateral Achilles vibration increases with the duration of vibration (10, 20 and 30 s) but the relationship is non-linear. The results of the present study showed that at least 16 s of vibration are necessary to induce most postural effects in young adults, regardless of the vibration condition. Less than 12 s of vibration[35, 36] is only sufficient to show the early effects of the proprioceptive perturbation. Results from studies involving stimulations of more than 20 s duration[3, 19] show adaptive sensory-motor effects which are not always taken into account in the analysis. During vibration at the elbow joint, Cordo et al.[18] showed that the illusion of movement disappears at around 16 s of vibration, whereas the illusion of position persists. It therefore appears that the halting of the postural displacement around this time is the result of central sensory adaptations rather than biomechanical regulation. Thus, the duration of the vibration has to be taken into account in the interpretation of mean or final CoP positions and in the design and methodology of studies.

## Conclusions

These results suggest that both the Triceps Surae and Peroneus muscles participate in the control of sagittal plane motion. The amplitude of the destabilization which occurs during vibration may be related to the functional role of the muscle in postural control. The proprioceptive perturbation of both the Triceps Surae and Peroneus Longus showed that proprioceptive information from these muscles plays a role in postural organization and integration within the motor pattern. Nevertheless, the results showed that the medial displacement of the CoP which occurred during unilateral Achilles vibration is not part of a general reaction to a single-limb perturbation. Proprioceptive input from the non-perturbed leg was not sufficient to avoid antero-posterior CoP displacement but helped to gain stability over time. This information could be useful for rehabilitation of the balance control process during walking. The results of this study showed that, even in a population of young subjects, 16 seconds of stimulation are necessary and sufficient to cause movement illusions and thus to destabilize subjects. These techniques could be helpful for the elaboration of therapeutic protocols.

## Author information

NCD is a physiotherapist and a PhD student. LM is a physiotherapist and the health manager of a center for neurological and orthopedic rehabilitation. JB is an associated professor. SM is a physiotherapist and an associated professor.

## Authors’ original submitted files for images

Below are the links to the authors’ original submitted files for images. Authors’ original file for figure 1 Authors’ original file for figure 2 Authors’ original file for figure 3 Authors’ original file for figure 4 Authors’ original file for figure 5 Authors’ original file for figure 6

## Abbreviations

CoP: Centre of pressure
AP: Antero-posterior
ML: Medio-lateral.
