# Supplementary figures and images for: Postural stabilization during bilateral and unilateral vibration of ankle muscles in the sagittal and frontal planes
Source: J Neuroeng Rehabil. 2014 Sep 1;11:130. doi: 10.1186/1743-0003-11-130 (PMC4162932; doi:10.1186/1743-0003-11-130)

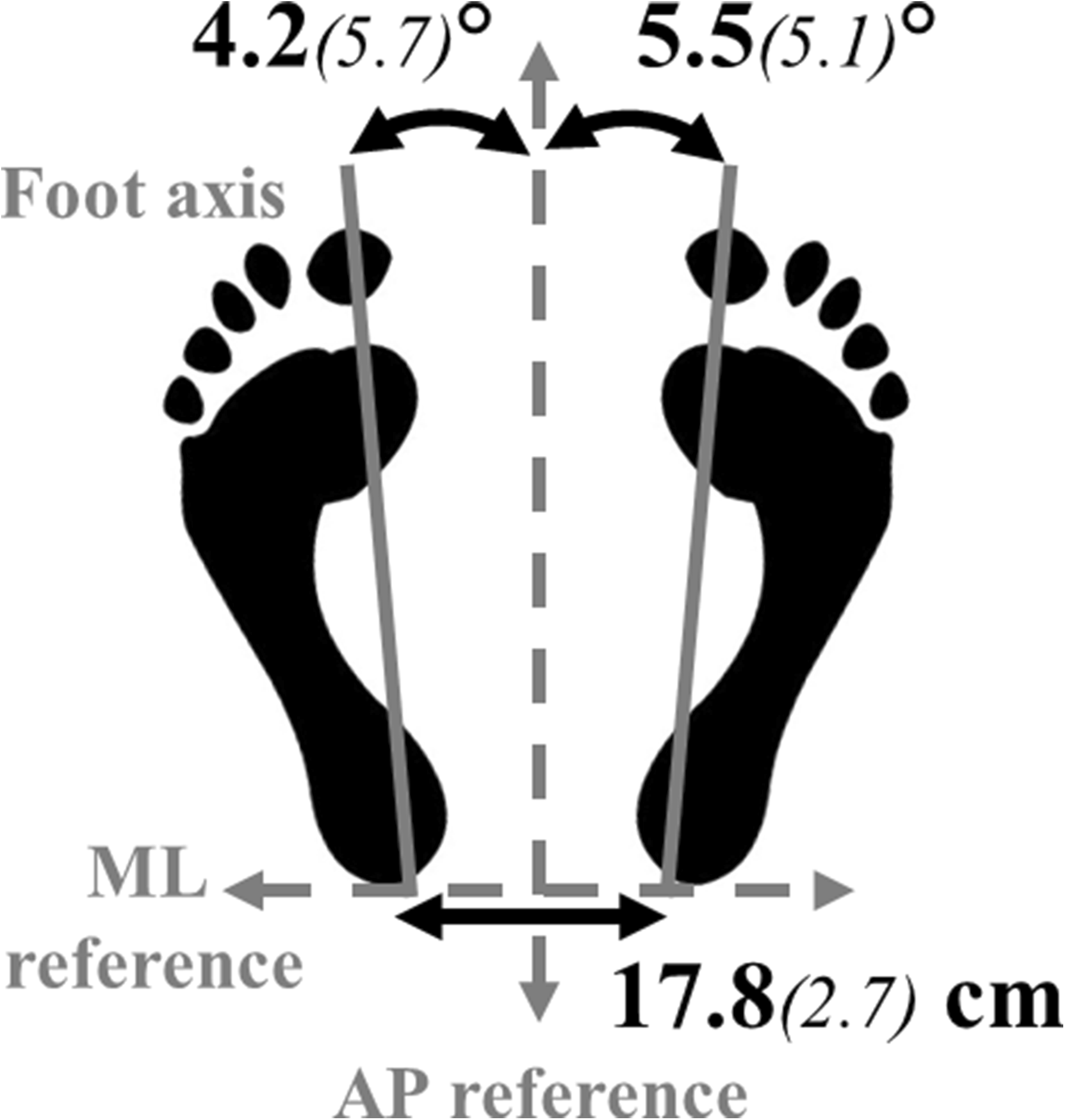

Supplement: Supplementary file 1 — Authors’ original file for figure 1 [file 12984_2014_652_MOESM1_ESM.tiff]

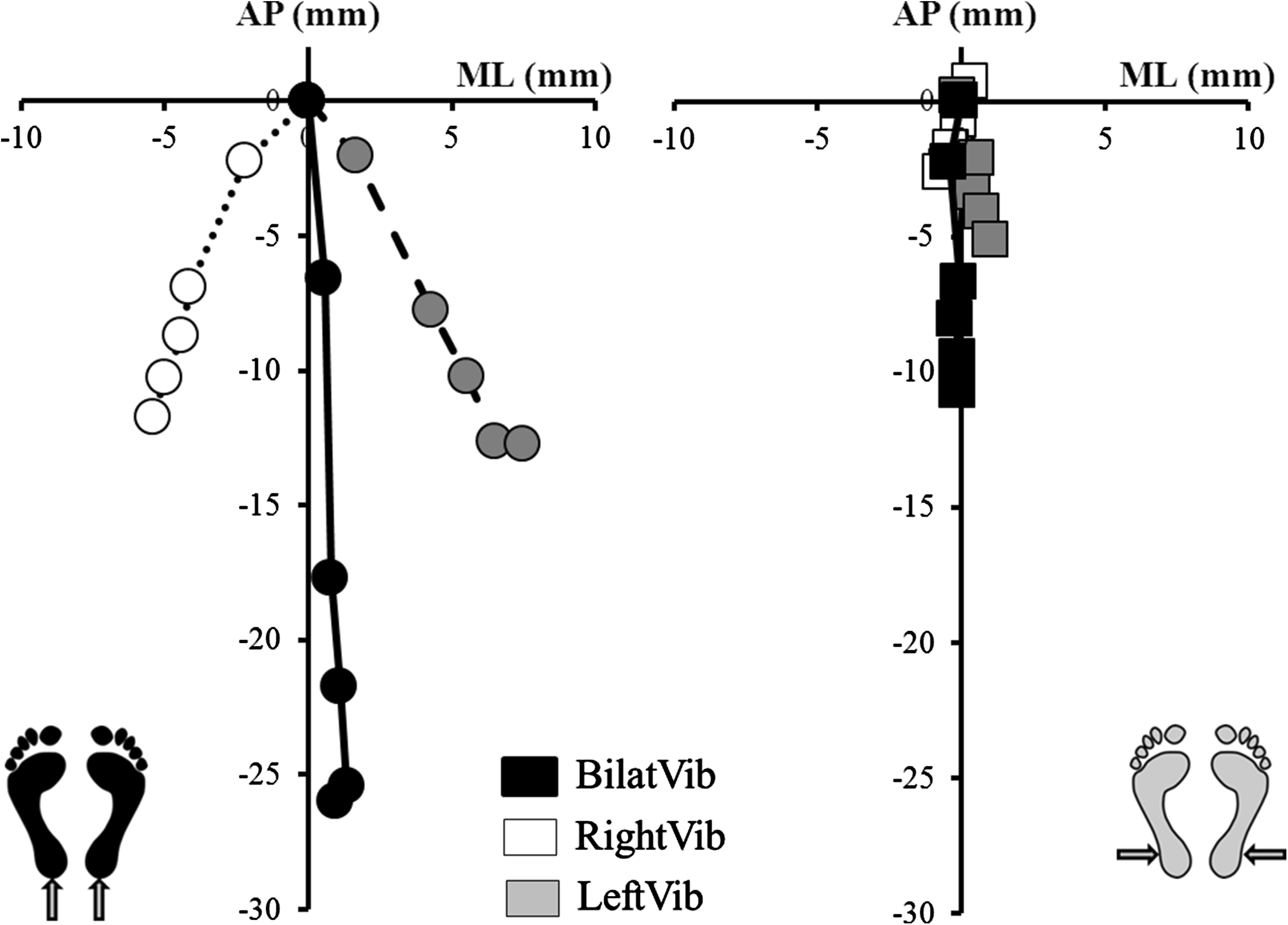

Supplement: Supplementary file 2 — Authors’ original file for figure 2 [file 12984_2014_652_MOESM2_ESM.tiff]

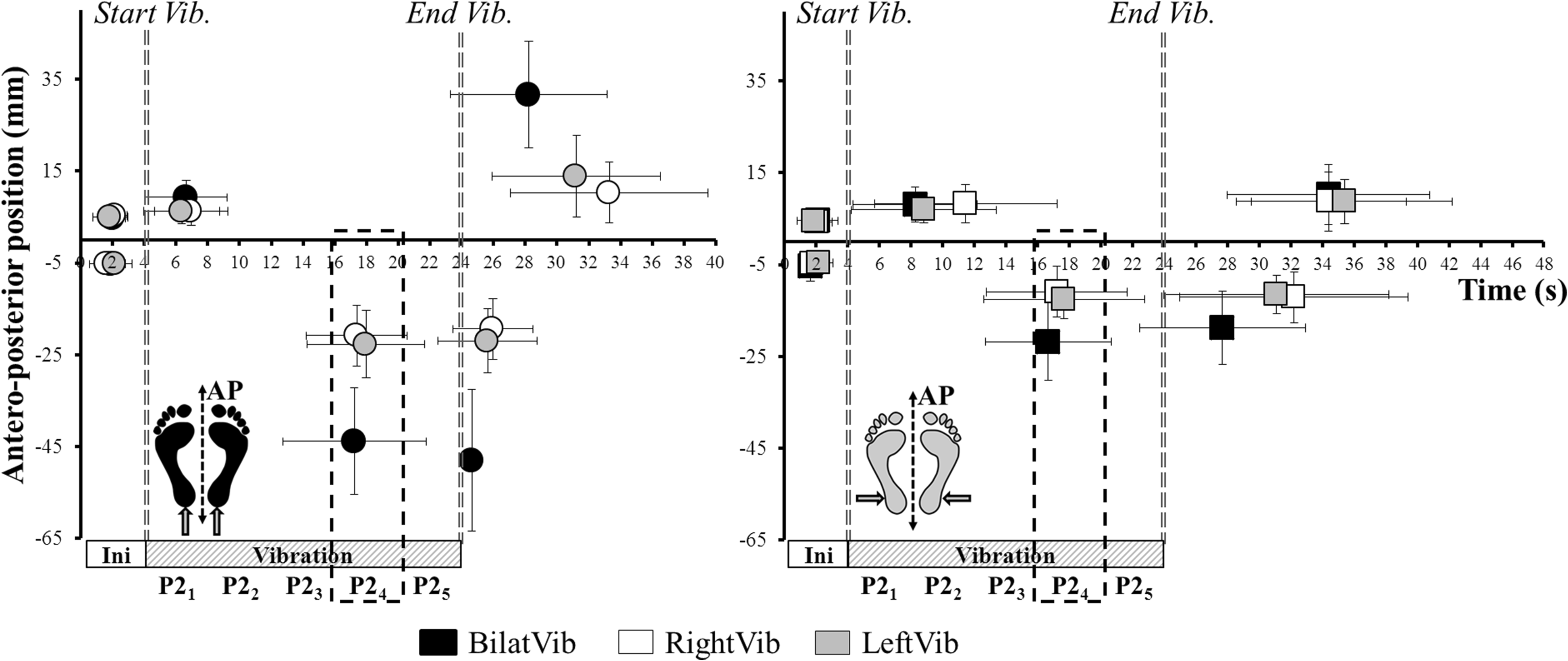

Supplement: Supplementary file 3 — Authors’ original file for figure 3 [file 12984_2014_652_MOESM3_ESM.tif]

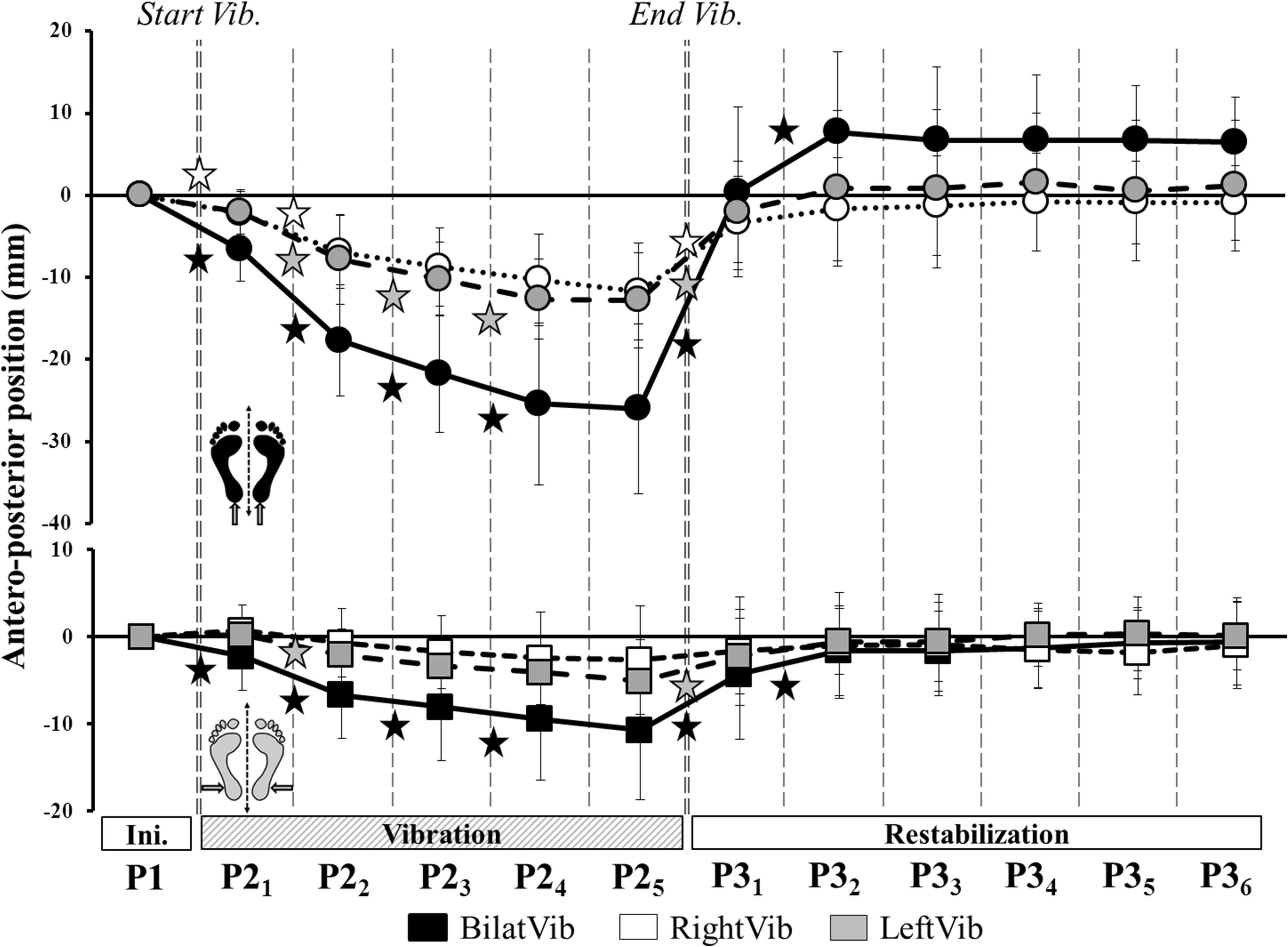

Supplement: Supplementary file 4 — Authors’ original file for figure 4 [file 12984_2014_652_MOESM4_ESM.tif]

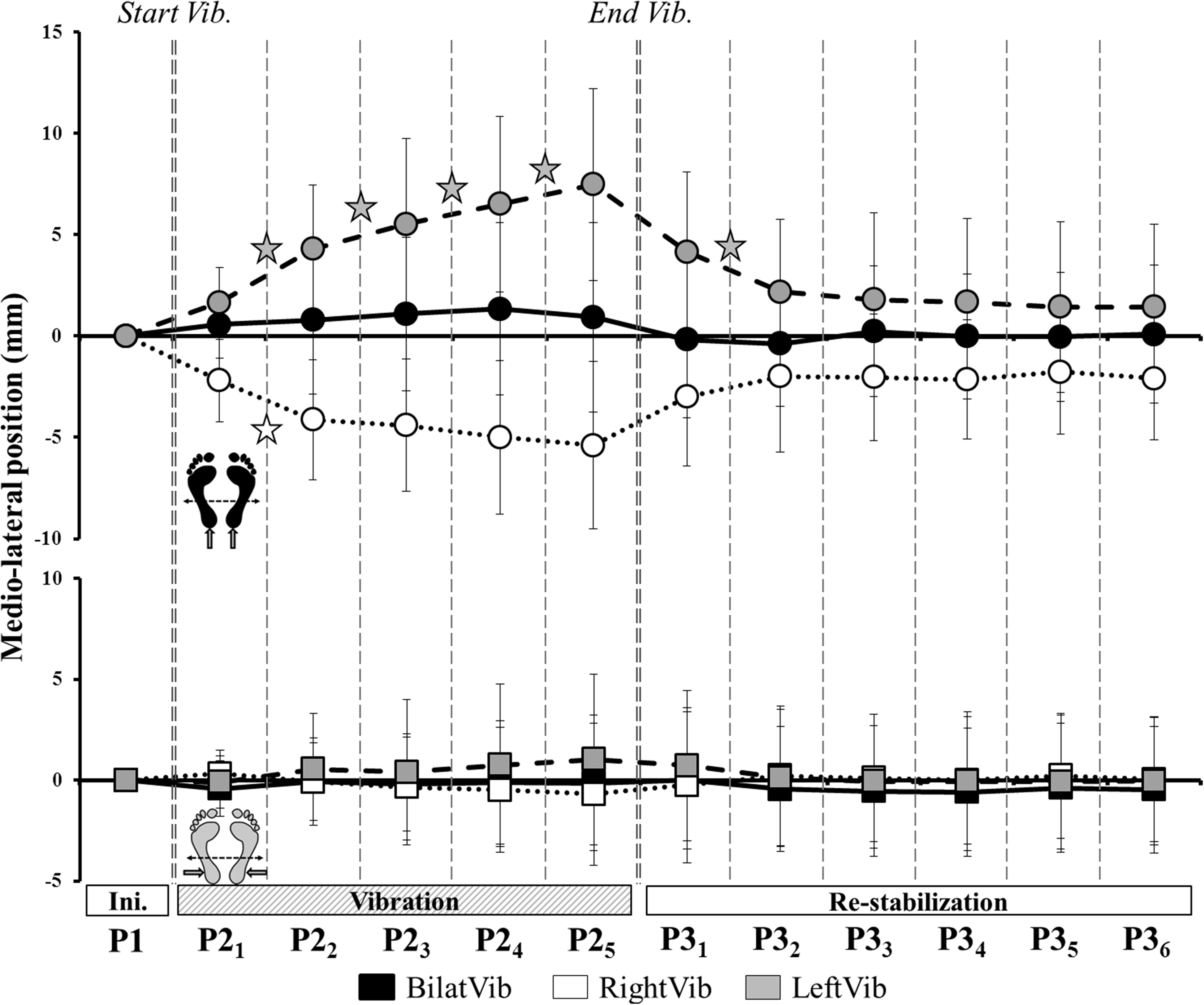

Supplement: Supplementary file 5 — Authors’ original file for figure 5 [file 12984_2014_652_MOESM5_ESM.tif]

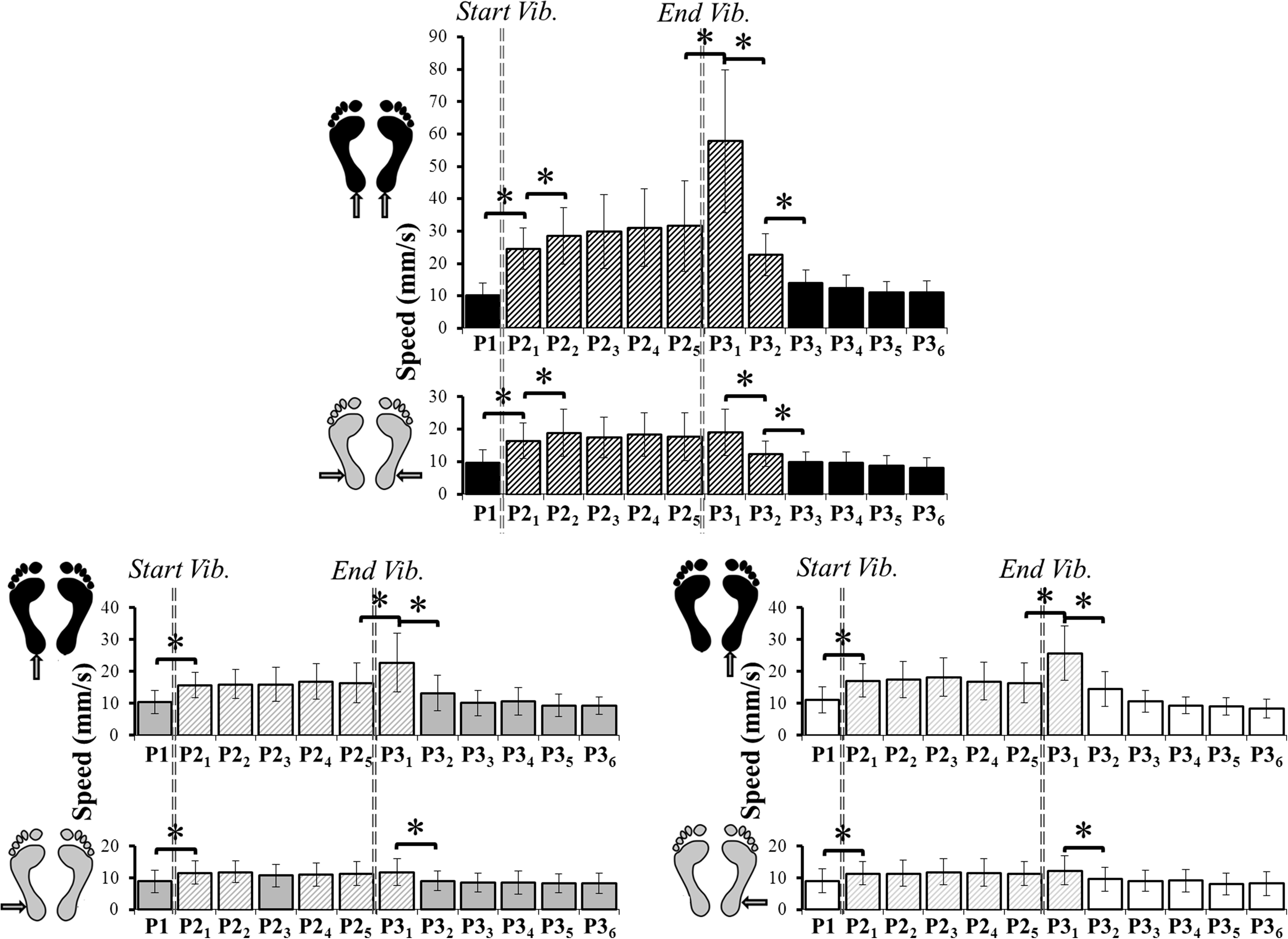

Supplement: Supplementary file 6 — Authors’ original file for figure 6 [file 12984_2014_652_MOESM6_ESM.tif]
